# Assessment of liver function tests of women taking hormonal contraceptives at University of Gondar comprehensive specialized hospital and Family Guidance Association of Gondar (FGAE), 2022; a comparative cross-sectional study

**DOI:** 10.1371/journal.pone.0289746

**Published:** 2023-08-17

**Authors:** Elias Chane, Habtamu Wondifraw, Rishan Hadgu, Alebachew Fasil

**Affiliations:** 1 Department of Clinical Chemistry, School of Biomedical and Laboratory Sciences, College of Medicine and Health Sciences, University of Gondar, Gondar, Ethiopia; 2 Department of Medical Laboratory Science, College of Medicine and Health Sciences, Arba Minch University, Arba Minch, Ethiopia; University of Health and Allied Sciences, GHANA

## Abstract

**Introduction:**

Hormonal contraceptives are artificial preparations that contain artificial progestins and Ethinylestradiol; these preparations are utilized by women of reproductive age to prevent pregnancy. Roughly a billion women in the world use some form of contraceptive worldwide. Despite the utility of these preparations, they are linked with several adverse effects, including disturbances of liver functionality and integrity. However, previous studies conducted to assess the association between hormonal contraceptive utilization and liver function tests reported conflicting results, and the effects remained a matter of concern.

**Methods:**

The study enrolled a total of 264 participants, who were allocated into two groups. One group of hormonal contraceptive users who use the medication for a minimum of six months: Depot medroxyprogesterone acetate (DMPA), combined oral contraceptives (COC), Norplant, and Implant users and another age-matched non-user control group in a ratio of 1:1. A semi-structured questionnaire was used to collect socio-demographic, behavioral, and clinical data. Five ml serum blood sample was collected for liver function test analysis on a Beckman Coulter Clinical Chemistry analyzer (DXC 700 AU). Independent t-test was used to compare liver function tests of hormonal contraceptive users and non-user controls, whereas ANOVA followed by a Bonferroni post hoc test was used for intra- (between classes of contraceptives) and inter-group (between each class of contraceptives and controls) comparisons and to identify factors associated.

**Results:**

Hormonal contraceptive users were observed to have a statistically significant higher mean value of liver enzymes assessed compared to non-user control groups: aspartate aminotransferase (AST) (47.07±14.79 versus 25.92±7.37; p <0.001), alanine aminotransferase (ALT) (35.83±13.76 versus 16.56 ± 5.03; p <0.001), alkaline phosphatase (ALP) (63.34±14.74 versus 45.41±14.34, p <0.001) and for γ-glutamyl transferase (GGT) (47.37±24.32 versus 19.45 ± 6.86 p <0.001). Similarly, the mean value of total and direct bilirubin (mg/dL) among HC users showed a statistically significant elevation (0.68 ± 0.22 against 0.32 ± 0.13, p <0.001) for total bilirubin and (0.14 ± 0.06 against 0.06 ± 0.03, p <0.001) for direct bilirubin respectively. However, no statistically significant result was observed in the mean values of total protein and albumin. For total protein (6.7 ± 0.89 versus 6.5 ± 1.15, p 0.07) and for albumin (5.4 ± 0.92 versus 5.3 ± 1.08; p 0.30). The current study also indicates the level of hepatic function test alteration is related to the type of hormonal contraceptives, duration of usage, and level of adherence to a specific class of contraceptives.

**Conclusion and recommendation:**

Hormonal contraceptive use was observed to affect hepatic function. Based on this finding, we strongly recommend to closely monitor liver function tests in women using hormonal contraceptives.

## Introduction

Hormonal contraceptives (HC) are preparations containing synthetic estrogen in the form of Ethinylestradiol (EE) and/or progestin (synthetic progesterone). These hormones commonly act on the endocrine system and allow sexual union without subsequent ovulation [1, 2]. Despite the utility of these preparations for the prevention of unplanned pregnancy, they are not without adverse effects. The most common adverse effects include metabolic impairment and cardiovascular (CV) complications [3]. Most of the adverse effects are related to the EE and progestin components of HC [4]. The metabolic effect of HCs affects several physiological processes and organs of the body, particularly the liver [5]. The EE component in HC is reported to increase the metabolic activities of the hepatocytes; on the other hand, larger doses of HC cause adaptative changes by increasing the level of metabolic enzymes [6, 7]. On the other hand, the principal constituents of HC, EE and progestin affect the metabolism of the body’s macromolecules in the liver [8].

Experimental studies showed that HCs cause time- and dose-dependent depletion of cytochrome P-450 [9–11]. Other studies also reported that HCs can induce liver damage, jaundice, and hepatocyte abnormalities [5, 12]. Animal studies have shown that certain structural features at the molecular level of HC could lead to structural and functional abnormalities of the liver [13]. These structures include a phenolic ring and 17 α-alkyl substitutions [14]. On the other hand, the most common methods of HCs worldwide, such as oral contraceptive pills (OC) and injectable Depot-Medroxyprogesterone Acetate (DMPA), have been shown to enhance oxidative stress (OS) [15–17]. This is due to the necessity to metabolize the hormone load from the HCs with the help of liver enzymes. [18].

Modern hormonal contraceptives are utilized by over 80% of women at some point in their reproductive years [19]. The data from “*World Fertility and Family Planning 2020*” state that in the year 2019, 1.9 billion women of reproductive age were living in the world, and 49% of all women of reproductive age were using some form of contraceptive globally [20] and half of women were using modern HCs [21]. Globally, the use of HC has risen slightly from 54% in 1990 to 57.4% in 2015 [22]. Modern HCs utilization has been increasing in many parts of the world [23]. The use of HC among women in sub-Saharan African nations improved from 13% in 1990 to 29% in 2019, and all other regions had a distribution of contraceptives of 50% or more [20]. In Ethiopia, the contraceptive prevalence rate (CPR) is currently 22.2% [24]. According to data from “*The 2019 Ethiopian Mini Demographic and Health Survey”* overall, 41% of currently married women are using methods of HCs [25].

About half of HC users stop using or change HC use within the first year of their prescription, mostly due to side-effects or concern about adverse health effects [26]. Menstrual cycle disturbance, irregular bleeding, increased body weight, and metabolic changes are the most frequent reasons for discontinuation of HCs [27, 28]. The other most frequent adverse effects reported with HC use are liver abnormalities [29, 30]. Previous studies have demonstrated a relationship between the use of HCs and cholestasis, hepatic neoplasms, and even vascular diseases [31]. Alterations can occur in certain liver tests among healthy women taking HC. This alteration may consist of abnormalities of plasma enzyme activity, plasma proteins, or even liver histology [5, 30].

Preeminent liver function tests (LFTs) are found in approximately 8% of the global population. A review on the prevalence of LFT abnormalities from a total of 37 studies reported that the general prevalence of atypical LFTs with at least one abnormal constituent in the LFT was high at 10–21.7% [32]. These elevations may be transient in patients without symptoms, with up to 30% of elevations resolving after 3 weeks [33]. The incidence of liver disease is increasing [34]. Liver diseases have been ranked as the 5^th^ most common cause of death worldwide [35].

In sub-Saharan Africa, cirrhosis-related deaths have doubled in the years between 1980 and 2010. During 2011, the estimated worldwide mortality from cirrhosis was over seven hundred thousand people, ranking 14^th^ and 10^th^ as the leading cause of death in the world and developed countries, respectively [34]. In Ethiopia, liver diseases accounted for 11.4% of all medical admissions. Viral hepatitis, post-hepatic or post-necrotic, mixed cirrhosis, and hepatocellular carcinoma were the different forms of liver disease reported [34, 36].

Several studies have been conducted worldwide to determine how various body organs and HC usage interact. However, little is known about liver function tests, and the few studies that have been conducted in this area show conflicting results. The questions about the effect of hormonal contraceptives on liver function tests remained unanswered. The liver metabolizes components of HCs, and these substances cause the liver to produce a change in its biological function [5]. However, the reported adverse effects of hormonal contraceptives are quite controversial and contradictory in previous literature. Most of these studies were conducted on a particular class of HCs, and the association has been shown to be inconsistent and remains controversial. Thus, there is a need to evaluate the real burden of the problem and come up with specific answers. As such, this study was undertaken to investigate the effects of HC on liver function tests among women on HC in Gondar town.

## Methods and materials

### Study setting, study design and period

The study was conducted at the University of Gondar Comprehensive Specialized Hospital (UoGCSH) family planning “*Mitchu*” clinic and Family Guidance Association (FGAE) of Gondar town in 2022. The institutions are located in the central Gondar administrative zone, Amhara regional state, which is about 741 kilometers northwest of Addis Ababa. A Comparative Cross-sectional study was conducted among hormonal contraceptive users and an age-matched non-user control group to assess and compare liver function tests. The study data was collected from March 2 to May 27, 2022.

### Study populations

The current study comprises a total of 264 participants enrolled in two comparable study groups. The first group comprised HC users (Norplant/Implant, DMPA, and oral contraceptive (OC) pills) and another age-matched non hormonal contraceptive user who visited UoGCSH and FGAE for routine family planning service and consultation at the time of data collection (March 2 to May 27, 2022) and fulfilled the inclusion criteria.

### Eligibility criteria

#### Inclusion criteria

All HC users who were using DMPA, oral contraceptive pills, Norplant, or Implant methods for a minimum of 6 months. Individuals who were visiting the two health facilities at the time of data collection, who voluntarily provided socio-demographic, behavioral, and clinical information, and those who were willing to provide 5 ml of serum blood sample were enrolled in HC user groups. Whereas women in the reproductive age group abstain from HC use, individuals who use non-hormonal contraceptives (Condoms, Cervical caps, and diaphragm) and individuals with a history of HC use but not in the last 6 months were considered controls. For all participants, a screening test for Hepatitis B, C and HIV/AIDS was performed; history of any liver disease was chalked from medical history chart. Blood FBS and blood pressure were measured to exclude individuals with metabolic syndrome.

#### Exclusion criteria

Exclusions included women with inconsistent contraceptive use, poor healthcare compliance, mental health problems, hearing impairments, and other health issues limiting their ability to provide information. Women on steroid or protein therapy, with PCOS, and with coexisting conditions affecting liver function tests were also excluded. Additionally, women taking medications known to affect liver function tests or with a history of pregnancy in the previous 24 months or breastfeeding were excluded.

### Sample size and sampling technique

The sample size was determined by using a double population proportion mean comparison. For sample size calculation, Open epi software version 2.3 was used. The values of the outcome variable were taken from the previous study conducted in Nigeria [37]. The sample size was calculated for each independent variables, and the calculation that gave the maximum sample size was considered. Taking the values of ALT for both HC users (24.5±10.7) and controls (17.4±5.6) gives the maximum sample size. Assuming a 95% confidence level, 80% power, and a 20% non-response rate, the minimal sample size in each group was calculated. This gives 56 participants were necessary for each study group. To maximize the power of the study, the number of study participants was increased two-fold for both study groups. Thus, a total of 264 (132 for HC user group and 132 for Non-user Controls) were enrolled in the study.

A systematic random sampling technique was applied to recruit participants. The sample size was allocated proportionally. According to data from the two institutes in the previous year, the expected number of individuals was calculated for the three-month duration of the study. Every 6^th^ of study participants arriving at the clinics were asked for participation and involved in the study from selected institutions.

### Data collection procedure

A standard questionnaire was prepared, and few questions were adapted from the “*WHO STEPS Questionnaire*” for behavioral and clinical characteristics.

The questionnaire was first prepared in English, and then it was translated into the local Amharic language. The questionnaire contains three portions: the first portion is about sociodemographic characteristics; the remaining two portions contain questions to assess the behavioral (Coffee drinking habit, Physical Exercise habits, dietary diversity score, usual food content, Smoking Habit, Alcohol drinking habits, Khat chewing habit) and clinical characteristics (Body mass index (BMI), waist-to-hip ratio (WHR), Blood Pressure) of participants; the prepared questions were pre-tested at the “*Maraki*” health center to adapt to the local context based on the study objectives through a face-to-face interview.

Individuals who fulfilled the inclusion criteria for each group of the study were asked to participate after being adequately informed about the objective of the study. Participants who agreed to participate were asked to provide written informed consent. Prior to actual data collection, the selected participants were screened for eligibility to study; fasting blood sugar (FBS), hepatitis B and C virus, and HIV testing were performed for both groups; HCG pregnancy test was performed for the control groups to rule out pregnancy. Participants who met the inclusion criteria were then interviewed about their sociodemographic, behavioral, and clinical characteristics.

Weight in kilograms (kg) was recorded to the nearest 0.1 Kg using a digital balance on a Seca digital weighting scale (Germany), with participants being barefoot and wearing light clothes. Height (cm) was measured twice and reported to the nearest 0.1 cm using a standard and calibrated Stadiometer Seca (Germany). BMI was calculated (kg/m^2^) using measured height and weight. The waist circumference was measured using a meter at the level midway between the lowest rib margin and the iliac crest. Hip circumference was measured using a meter around the widest part of the hips, crossing behind the back. Blood pressure (BP) was measured using an analog sphygmomanometer (Omron blood pressure monitor Japan) and stethoscope (Omron Japan). Measurements were taken from the upper arm while placing the hand at the heart level after the participants had sat for more than 5 minutes. Systolic blood pressure ≥140 mmHg and/or diastolic blood pressure ≥90 mmHg were used to define hypertension.

After the interview, the upper arm of the hand was disinfected using 70% alcohol swaps in a circular motion from inside to outside fashion and allowed to dry completely. Then, the needle was entering swiftly at a 30-degree angle. A fasting venous blood sample of 5 milliliters (ml) was collected from the medial cubital vein of the left arm with a 19-gauge syringe and then transferred to a jell-coated serum separator tube (SST); the tube was labeled with a unique ID number. The blood was left to form a blood clot at room temperature for 30 minutes, then centrifuged at 3500 RPM for 5 minutes. The serum was transferred to a sterile “Nunc tube” and stored in a refrigerator at 2–8°C until analysis.

### Laboratory procedure

Liver function test (LFT) was analyzed from serum using Beckman coulter Dxc700 Automated Chemistry Analyzer DxC 700 AU clinical chemistry analyzer is the latest innovation manufactured by Beckman Coulter Inc. is a Danaher Corporation company Brea, California, United States. Enzymatic LFT results were reported as IU/L, and the other LFT results were reported in mg/dl.

Serum AST activity was measured by the method, in which AST catalyzes the transamination of aspartate and α-oxoglutarate, forming L-glutamate and oxalacetate. The oxalacetate is then reduced to L-malate by malate dehydrogenase, while NADH is simultaneously converted to NAD+. The decrease in absorbance due to the consumption of NADH is measured at 340 nm and is proportional to the AST activity in the sample [38].

Serum ALT activity was measured by the method, in which ALT transfers the amino group from alanine to α-oxoglutarate to form pyruvate and glutamate. The pyruvate enters a lactate dehydrogenase (LD) catalyzed reaction with NADH to produce lactate and NAD+. The decrease in absorbance due to the consumption of NADH is measured at 340nm and is proportional to the ALT activity in the sample [38].

Serum ALP activity is determined by measuring the rate of conversion of p-nitro-phenylphosphate (pNPP) in the presence of 2-amino-2-methyl-1-propanol (AMP) at pH 10.4. The rate of change in absorbance due to the formation of pNPP is measured bichromatically at 410/480 nm and is directly proportional to the ALP activity in the sample.

Total Bilirubin was measured using the classical method. A stabilized diazonium salt, 3,5-dichlorophenyldiazonium tetrafluoroborate (DPD), reacts with bilirubin to form azobilirubin, which absorbs at 570/660 nm. Caffeine and a surfactant are used as reaction accelerators. The absorbance at 570/660 nm is proportional to the bilirubin concentration in the sample. A separate serum blank is performed to eliminate endogenous serum interference [38].

Direct Bilirubin was measured using a variation of the classical method. Direct (conjugated) bilirubin couples directly with a diazonium salt of 3,5-dichloroaniline (DPD) in an acid medium to form azobilirubin. The direct bilirubin in serum is directly proportional to the color development of azobilirubin, which is measured bichromatically at 570/660 nm.

GGT was measured enzymatically based on the principle that GGT catalyzes the transfer of the gamma-glutamyl group from the substrate, gamma-glutamyl-3-carboxy-4-nitroanilide, to glycylglycine, yielding 5-amino-2-nitrobenzoate. The change in absorbance at 410/480 nm is due to the formation of 5-amino-2-nitrobenzoate and is directly proportional to the GGT activity in the sample.

Total protein was measured by the method in which Cupric ions in an alkaline solution react with proteins and polypeptides containing at least two peptide bonds to produce a violet-colored complex. The absorbance of the complex at 540/660 nm is directly proportional to the concentration of protein in the sample.

Serum albumin was measured at pH 4.2; bromocresol green reacts with albumin to form an intense green complex. The absorbance of the albumin BCG complex is measured bichromatically (600/800nm) and is proportional to the albumin concentration in the sample.

### Data quality control

Prior to actual data collection, the prepared questionnaires were translated from English to Amharic and pretested in Maraki Health Center on about 5% of the sample size (15 individuals) to assure their accuracy and consistency. Training was given to data collectors about the objective and relevance of the study, confidentiality issues, study participants’ rights, consenting, techniques of interviewing, laboratory test procedures, and their quality control. The blood sample collection and laboratory tests were performed by well-trained nurses and laboratory technologists. The collected samples were analyzed by Beckman coulter Dxc 700 chemistry analyzer, their quality was checked by previously documented Amhara regional state, Regional Laboratory, and EQA sample result feedback reports.

Standard quality control (normal and pathological) protocols were performed and assured before running the participants’ samples to assure the analytical performance, accuracy, and functionality of the instrument. Furthermore, the investigator closely followed and frequently checked the sample analysis process to ensure its completeness and consistency.

### Data analysis and interpretation

The collected data was checked, sorted, categorized and, coded manually. Data was reviewed daily to assure appropriateness, avoid any identifier, and ensure confidentiality unique participant code was used. The data was entered into EPI-DATA (version 4.6) software. The data was cleaned, transferred into STATA VERSION 14, and analyzed for descriptive statistics. The normal distribution of continuous variables was checked using kolmogorov-smirnov test. Independent t-test and ANOVA with Bonferroni post hoc test was used to compare the values among each group and to identify associated factors respectively.

### Ethical consideration

Ethical clearance was obtained from research and ethical review committee of the School of Biomedical and Laboratory Sciences, College of Medicine and Health Sciences University of Gondar. Ethics approval number for the study; Ref No. SBMLS/184/2022 the study was conducted following the Declaration of Helsinki. A permission letter was also obtained UoGCSH medical director, School of Midwifery, and head of Medical Laboratory. Another Permission letter was also taken from the clinical director and head of FGAE. All study data was collected after informed consent was given by each participant. All participants provided written and signed informed consent to participate and provide their data to this study. To ensure confidentiality of the study participant’s information, codes was used so that the name and any identifier of participants were not used on the questionnaire and laboratory requests. The collected data was not used for another purpose other than the present study. Participants with abnormal test results were communicated through data collectors and linked to University of Gondar Comprehensive Specialized Hospital for treatment purpose.

## Results

### Sociodemographic characteristics of study participants

A total of 264 study participants were included in the present study, of which 132 were HC-users and the remaining 132 were age-matched controls. The mean ages of the two groups were 26.75 (±5.40) for HC-users and 26.69 (± 5.54) for controls. The majority of study participants, 87 (65.91%) of HC-users and 82 (62.12%) of the controls, were aged between 21–29 years. Majority of the study participants 209 (79.17%) were married. 126 (95.45%) of HC- user groups dwell in the urban part of the Gondar, whereas 120 (90.91%) of controls were from urban areas (**Table 1**).

**Table 1:** Sociodemographic characteristics among hormonal contraceptive users and non-user controls participants (n = 264, Gondar, 2022).

Variables	Categories	HC-users	Controls	Total	p-values
		n (%)	n (%)	n (%)	
**Age group**	≤ 20	10 (7.58%)	12 (9.09%)	22 (8.33%)	0.91
	21–29	87 (65.91%)	82 (62.12%)	169 (64.0%)	
	30–43	35 (26.51%)	38 (28.79%)	73 (27.6%)	
**Monthly Income**	≤1500 ETB	79 (59.85%)	70 (53.03%)	149 (56.4%)	0.26
	>1500 ETB	53 (40.15%)	62 (46.97%)	115 (43.5%)	
**Educational Status**	Unable to read/write	21 (15.91%)	27 (16.67%)	48 (18.18%)	0.04*
	Primary School	29 (21.91%)	11 (8.33%)	40 (15.15%)	
	High School	52 (39.39%)	57 (43.18%)	109 (41.29%)	
	Higher Education	30 (22.73%)	37 (28.03%)	67 (25.38%)	
**Residence**	Urban	126 (95.45%)	120 (90.91%)	246 (93.18%)	0.14
	Rural	6 (4.55%)	12 (9.09%)	18 (6.82%)	
**Marital Status**	Single	15 (11.36%)	31 (23.48%)	46 (17.42%)	NA
	Married	114 (86.36%)	95 (71.92%)	209 (79.17%)	
	Widowed	-	1 (0.76%)	1 (0.38%)	
	Divorced	3 (2.27%)	5 (3.79%)	8 (3.03%)	
**Occupation**	House wife	61 (46.21%)	45 (34.09%)	106 (40.15%)	0.007 *
	Gov’t employee	9 (6.82%)	15 (11.36%)	24 (9.09%)	
	Merchant	26 (19.70%)	17 (12.88%)	43 (16.29%)	
	Student	17 (12.88%)	44 (33.33%)	61 (23.11%)	
	Daily labor	13 (9.85%)	8 (6.06%)	21 (7.95%)	
	Other ^**#**^	6 (4.55%)	3 (2828%)	9 (3.40%)	

^**#**^ Servant, private-employee, farmer & unemployed

* Statistically significant difference on chi^2^ test

ETB- Ethiopian Birr (1 ETB equals 0.018 USD (Ethiopian National Bank, July 2023))

### Clinical and behavioral characteristics of participants

Among a total of 264 study participants, the mean body mass index (BMI) of the HC-user group was 22.73 (± 4.12) and 22.44 (± 3.83) for non-user controls. The majority of HC-users 113 (85.61%) and 119 (90.15%) of controls had mean systolic blood pressure (SBP) of < 120 mmHg. About 110 (83.33%) of HC-users and 114 (86.36%) of controls had mean fasting blood sugar (FBS) values less than 100 mg/dl. The majority of the HC-users 100 (75.76%) and 113 (85.61%) of non-HC user controls experience fewer than 2 pregnancies throughout their lives. 107 (81.06%) HC-users and 100 (75.76%) controls had coffee drinking habit. Plants/vegetables were observed to be the most common food contents in both groups 99 (75.0%) and 90 (68.18%) among HC-users and controls, respectively (**Table 2**).

**Table 2:** Clinical and behavioral characteristics among hormonal contraceptive users and non-user control participants (n = 264, Gondar, 2022).

Variables	Categories		HC users	Controls	Total	P-values
			n (%)	n (%)	n (%)	
**BMI (Kg/m** ^**2**^ **)**	Under weight (< 18.5)		18 (13.64%)	17 (12.88%)	35 (13.2%)	0.83
	Normal (18.5–24.99)		76 (57.58%)	83 (62.88%)	159 (60.2%)	
	Over weight (25–30)		30 (22.73%)	25 (18.94%)	55 (20.8%)	
	Obese (> 30)		8 (6.06%)	7 (5.30%)	15 (5.68%)	
**WHR**	≤ 0.85		65 (49.24%)	76 (57.58%)	141 (53.4%)	0.17
	> 0.85		67 (50.76%)	56 (42.42%)	123 (46.6%)	
**FBS (mg/dl)**	≤ 100 mg/dl		110 (83.33)	114 (86.36%)	224 (84.8%)	0.49
	>100 mg/dl		22 (16.67%)	18 (13.64%)	40 (15.15%)	
**Hgb (g/dl)**	< 12 g/dl		27 (20.45%)	42 (31.81%)	69 (26.1%)	0.036 *
	≥12 g/dl		105 (79.5%)	90 (68.19%)	195 (73.9%)	
**Blood Pressure(mmHg)**	SBP	≤ 120 mmhg	113 (85.61%)	119 (90.15%)	232 (87.8%)	0.25
		>120 mmhg	19 (14.39%)	13 (9.85%)	32 (12.2%)	
	DBP	≤ 80 mmhg	123 (93.18%)	127 (96.21%)	250 (94.6%)	0.56
		> 80 mmhg	9 (6.82)	5 (3.94%)	14 (5.4%)	
**Family history of DM**	Yes		23 (17.42%)	8 (6.06%)	31 (11.8%)	0.004 *
	No		109 (82.58%)	124 (93.94%)	233 (88.2%)	
**Family history of HTN**	Yes		25 (18.94%)	13 (9.85%)	38 (14.39%)	0.03*
	No		107 (81.06%)	119 (90.15%)	226 (85.6%)	
**Number of Pregnancy**	Zero		34 (25.75%)	52 (39.39%)	86 (32.58%)	0.02*
	≤ 2 Times		66 (50.00%)	61 (46.21%)	127 (48.1%)	
	> 2 times		32 (24.24%)	19 (14.39%)	51 (15.6%)	
**Parity**	Nulliparous		9 (6.81%)	13 (9.84%)	22 (8.3%)	0.24
	One or two		63 (47.7%)	50 (37.8%)	113 (42.8%)	
	Three or above		60 (45.45%)	69 (52.27%)	129 (48.8%)	
**Coffee Drinking(Cup/ day)**	No		25 (18.94%)	32 (24.24%)	57 (21.59%)	0.001*
	<2 cups per day		23 (17.42%)	6 (4.54%)	29 (10.9%)	
	≥2 cups per day		84 (63.64%)	94 (71.21%)	178 (67.42%)	
**Physical Exercise habit**	No (Inactive)		67 (50.76%)	97 (73.48%)	164 (62.1%)	0.73
	≤ 3 Days/week		9 (6.81%)	4 (3.48%)	13 (4.9%)	
	>3 days/week		56 (42.42%)	31 (23.48%)	87 (32.9%)	
**Eating Healthy food contents(High dietary diversity)**	No		5 (3.79%)	9 (6.82%)	14 (5.3%)	0.001*
	Sometimes		66 (50.0%)	87 (65.91%)	153 (57.9%)	
	Usually.		60 (45.45%)	31 (23.48%)	91 (34.46%)	
	Always		1 (0.76%)	5 (3.79%)	6 (2.27%)	
**Usual Food content**	Plant /Vegetables sources		99 (75.0%)	90 (68.18%)	189 (71.6%)	0.21
	Animals and dairy products		33 (25.0%)	42 (31.82%)	75 (28.4%)	
**Smoking Habit**	No		131 (99.24%)	132 (100%)	263 (99.6%)	NA
	Yes		1 (0.76%)	-	1 (0.38%)	
**Alcohol drinking**	Yes		23 (17.42%)	33 (25.00%)	56 (21.21%)	0.13
	No		109 (82.58%)	99 (75.00%)	208 (78.8%)	
**Khat chewing**	Yes		8 (6.06%)	2 (1.52%)	10 (3.78%)	NA
	No		124 (93.94%)	130 (98.58)	254 (96.2%)	
**Underlining medical conditions**	Absence		111 (84.09%)	132 (100%)	*243 (92*.*04%)*	NA
	Chronic Migraines		4 (3.03%)	*-*	*4 (1*.*51%)*	
	Kidney problem		2 (1.51%)	*-*	*2 (0*.*75%)*	
	Gastritis		12 (9.09%)	*-*	*12 (4*.*54%)*	
	Epilepsy		3 (2.27%)	*-*	*3 (1*.*13%)*	

* Statistically significant difference between groups **NA**- not applicable

### Hormonal contraceptive status of participants

**Fig 1:**
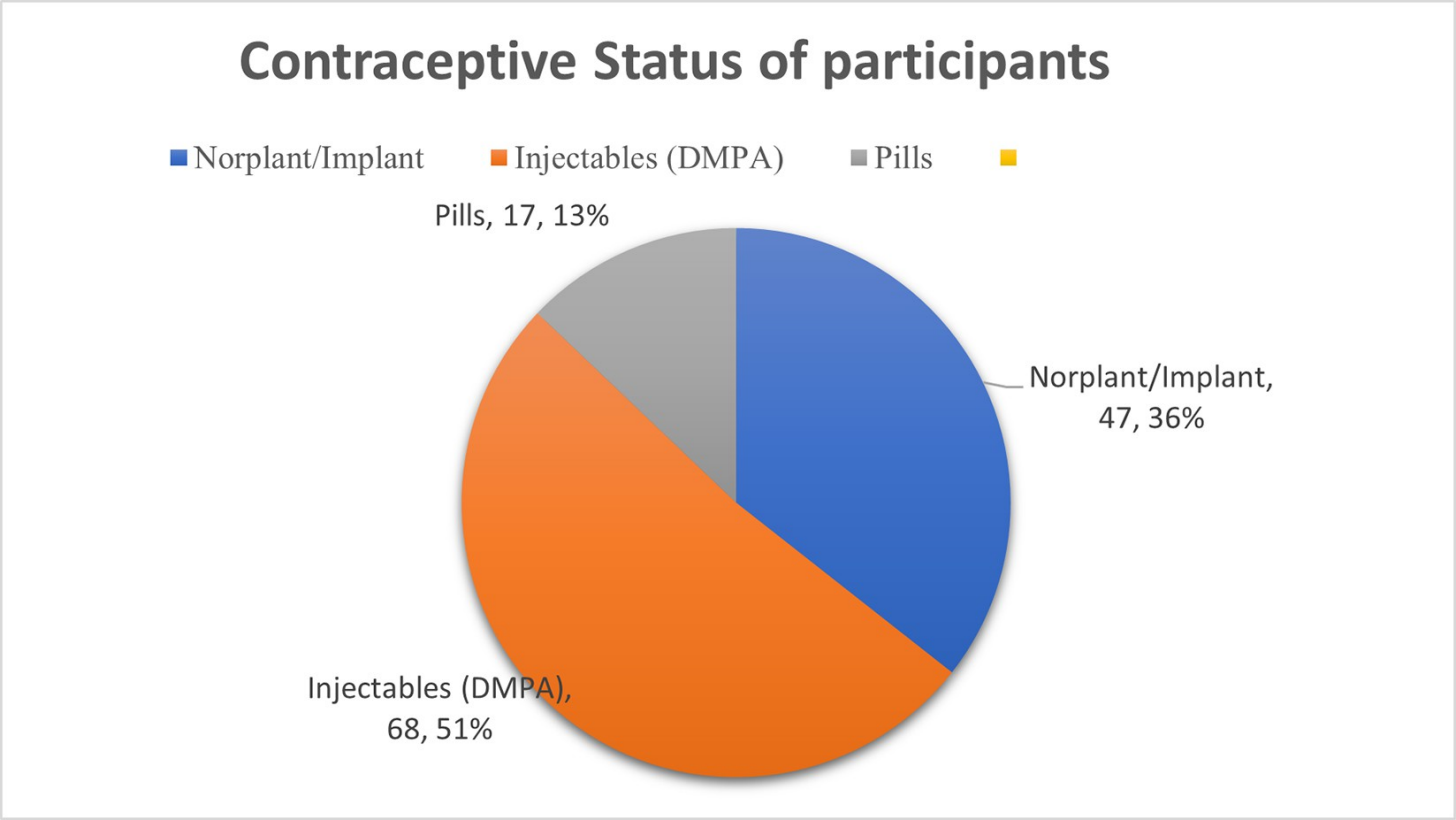
Contraceptive status of study participants.

**Fig 2:**
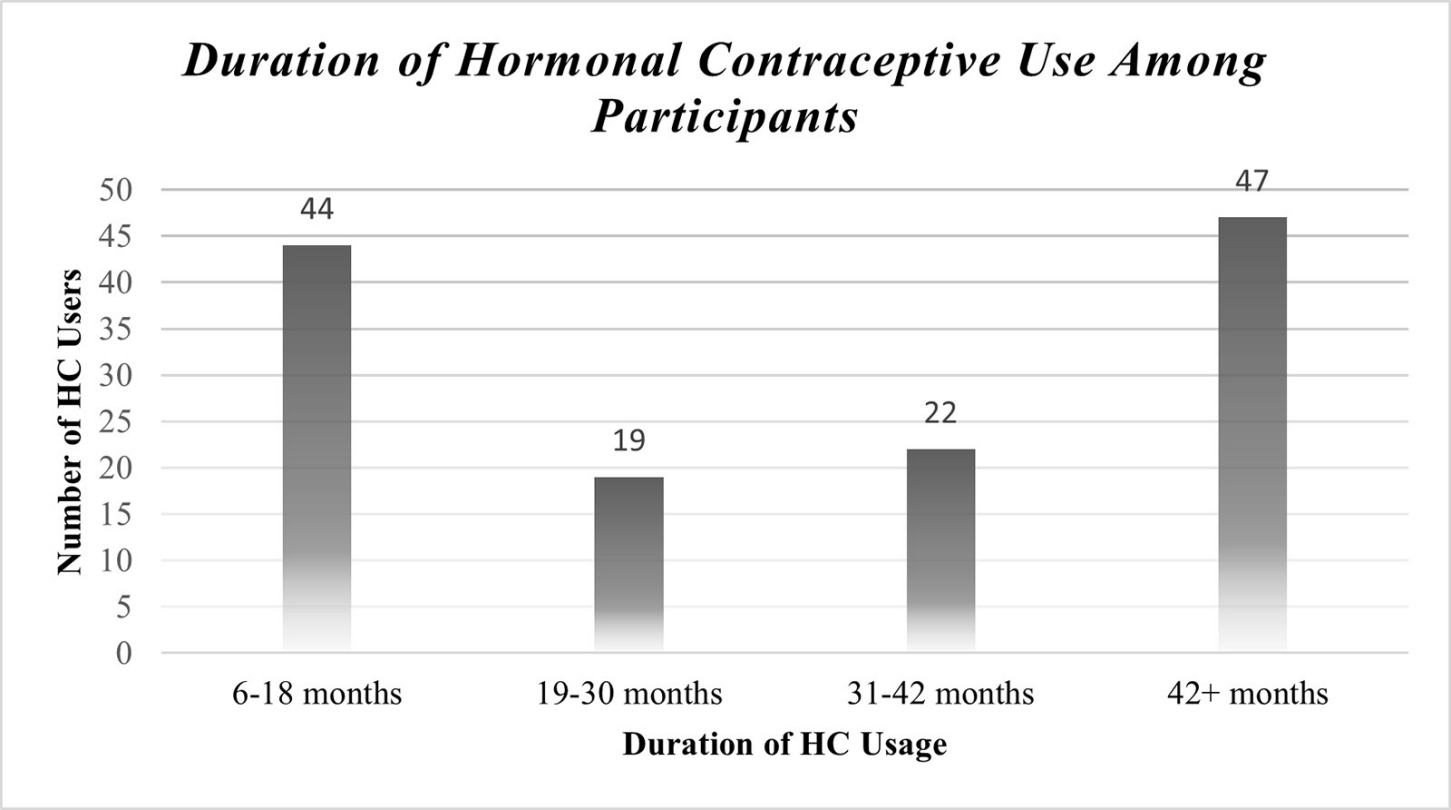
Duration of hormonal contraceptive utilization.

Most of HC user participants 68 (51.52%) were on Depot Medroxyprogesterone Acetate (DMPA) Whereas 47 (35.61%) were on Norplant/Implant. Most of the HC users 47 (35.61%) utilize HC methods for more than 42 months (3.5 years) (**Figs 1 & 2).**

About 79 (59.8%) changed their initial HCs method at least once, either due to experience of side-effects 68 (51.51%) or to prolong their regime time 11 (8.33%). About 44 (33.33%) of HC users utilize birth control methods for a short period of time (< 18 months).

### Comparison of liver function tests among participants

In the present study HC-user group, showed a statistically significant (p<0.05) higher mean value of liver enzymes (IU/L) assessed compared to non-user control groups: AST (47.07± 14.79 versus 25.92±7.37; p <0.001), ALT (35.83±13.76 versus 16.56 ± 5.03; p <0.001), ALP (63.34±14.74 versus 45.41±14.34, p <0.001) and GGT (47.37±24.32 versus 19.45 ± 6.86 p <0.001) (**Table 3**).

**Table 3:** Comparison of liver function tests among hormonal contraceptive users and non-user controls (n = 264, Gondar 2022).

Parameters	HC- Users	Controls	Mean difference (%)	P-value
	x±SD	x±SD		
**AST (IU/L)**	47.07 (± 14.79)	25.92 (±7.3)	+21.15 (81.59%)	< 0.001*
**ALT (IU/L)**	35.83 (±13.76)	16.56 (±5.03)	+19.27 (116.3%)	< 0.001 *
***De Ritis ratio* (AST/ALT)**	1.37 (± 0.31)	1.66 (± 0.57)	- 0.29 (21.16%)	< 0.001*
**ALP (IU/L)**	63.34 (± 14.74)	45.41 (±14.34)	+17.93 (39.3%)	< 0.001*
**T-Bilirubin (mg/dl)**	0.68 (± 0.22)	0.32 (± 0.13)	+0.36 (112.5%)	< 0.001*
**D-Bilirubin (mg/dl)**	0.14 (± 0.06)	0.06 (± 0.03)	+0.08 (113.3%)	< 0.001*
**Total Protein (g/dl)**	6.7 (± 0.89)	6.5 (± 1.15)	+0.2 (3.07%)	0.07
**Albumin (g/dl)**	5.4 (± 0.92)	5.3 (± 1.08)	+0.1 (1.88%)	0.30
**GGT (IU/L)**	47.37 (± 24.32)	19.45 (± 6.86)	+27.92 (143.5%)	< 0.001*

*Statistically significant by independent t-test is used for comparison

“+” Increase among Hormonal contraceptive users “-” Decreased among Hormonal contraceptive users

“T-Bilirubin”–Total Bilirubin “D-Bilirubin”–Direct Bilirubin

### Factor associated with alteration of liver function tests

#### Effect of classes of hormonal contraceptive on liver function tests

Different classes of HC were observed to have quite different effects on the alteration of LFT in study groups as shown on the ANOVA table below. All the assessed liver enzymes (AST, ALT, ALP, and GGT) showed a significant mean difference (p<0.05) between the three contraceptive classes (Norplant/Implant, DMPA, and COC) and control groups. A similar result obtained for total and direct bilirubin, with a statistical significance result (P< 0.001).

Bonferroni post hoc showed that the mean serum level of LFT among control groups was shown to be significantly lower than each class of contraceptive users (p < 0.001). On the other hand, the mean AST level of COC (Pill) users were observed to be significantly higher than the mean value than Norplant/Implant users (p< 0.05); Conversely, AST and ALP level of DMPA users were observed to be significantly higher than mean AST and ALP mean value of Norplant/Implant users (p <0.001). COC(Pill) users observed to have a statistically significant higher mean total bilirubin than Norplant users (p <0.001); DMPA users observed to have a statistically significant higher mean value for Total and direct bilirubin than Norplant/Implant users (p < 0.05). COC (Pill) users showed to have a statistically significant higher mean GGT level than Norplant/Implant users in a post hoc Bonferroni test (p <0.001) (**Table 4**).

**Table 4:** Effect of class of hormonal contraceptive on liver function tests among HC-users and non-user controls (n = 264, Gondar, 2022).

Parameters	Type /class of HCs				
	Norplant/Implant Users	DMPA (Injectable) users	Pill (COC) users	Controls	P-value
	x±SD	x+SD	x+SD	x+SD	
**AST (IU/L)**	43.29 (±12.62)	48.73 (±16.03)	50.88 (±13.73)	25.92 (±7.37) ^a^	< 0.001*
**ALT (IU/L)**	30.7 (±11.67)	37.30 (±14.49) ^c^	44.11 (±10.94) ^b^	16.56 (±5.03) ^a^	< 0.001*
**ALP (IU/L)**	57.04 (±13.71)	66.75 (±14.42) ^c^	67.11 (±13.60)	45.41 (±14.34) ^a^	0.03*
**T-Bilirubin (mg/dl)**	0.60 (±0.13)	0.71 (± 0.26) ^c^	0.80 (±0.22) ^b^	0.32 (± 0.13) ^a^	< 0.001*
**D-Bilirubin (mg/dl)**	0.12 (± 0.04)	0.160 (± 0.07) ^c^	0.164 (± 0.06)	0.06 (± 0.03) ^a^	<0.001*
**GGT (IU/L)**	43.29 (± 22.22)	45.19 (± 24.46)	67.35 (±20.58) ^b^^d^	19.45 (±6.86) ^a^	< 0.001*

*Statistically significant in One way ANOVA

a-d Superscript letters represent statistical significance in post hoc Bonferroni post hoc test

“T-Bilirubin”–Total Bilirubin “D-Bilirubin”–Direct Bilirubin

p <0.001 when Control groups compared to all HC user groups (Norplant/Implant, DMPA & pill (COC)) (One-way ANOVA/Bonferroni post hoc test)

p <0.001 when COC (pill) user group compared to Norplant/Implant users’ group

(One-way ANOVA/Bonferroni post hoc test)

p <0.001 when DMPA users groups compared to Norplant/Implant users’ group

(One-way ANOVA/Bonferroni post hoc test)

p <0.001 when COC (pill) user groups compared to DMPA users’ group

(One-way ANOVA/Bonferroni post hoc test)

#### Effect of duration of hormonal contraceptive use on liver function tests

It was observed that the mean level of ALP showed a statistically significant (p 0.003) difference when comparison is made through the duration of HC- use. On Bonferroni post-hoc test, it was observed that individuals who use hormonal contraceptives for 31–42 months had a lower mean ALP value than the other duration groups (**Table 5**).

**Table 5:** Effect of duration of hormonal contraceptive use on liver function tests among HC-users group (n = 132, Gondar, 2022).

Parameters	Duration of HC Use				
	6–18 Months (n = 44)	18–30 Months (n = 19)	30–42 Months (n = 22)	42 + Months (n = 47)	P-value
	x±SD	x±SD	x±SD	x±SD	
**AST (IU/L)**	51.04 (± 14.94)	46 (±14.54)	41.5 (±12.53)	46.40 (±15.15)	0.08
**ALT (IU/L)**	38.45 (±14.63)	36.94 (±14.40)	29.90 (±8.87)	35.70 (±14.09)	0.12
**ALP (IU/L)**	68.02 (±14.88)	62.94 (±13.28)	53.95 (±10.67) ^a^	63.51 (±15.08)	0.0031*
**T- Bilirubin (mg/dl)**	0.71 (±0.21)	0.71 (±0.18)	0.60 (± 0.15)	0.69 (± 0.27)	0.25
**D- Bilirubin (mg/dl)**	0.16 (± 0.06)	0.13 (± 0.05)	0.13 (± 0.05)	0.15 (± 0.07)	0.18
**GGT(IU/L)**	50.86 (± 27.23)	50.47 (± 24.98)	35.13 (± 14.53)	48.57 (± 23.70)	0.07

* Statistically significant in One way ANOVA

“T-Bilirubin”–Total Bilirubin “D-Bilirubin”–Direct Bilirubin

a- p <0.05 when HC users’ group of 31–42 months compared to users’ group of 6–18 months

(One-way ANOVA/Bonferroni post hoc test)

#### Effect of changing hormonal contraceptive method on liver function tests

**Fig 3:**
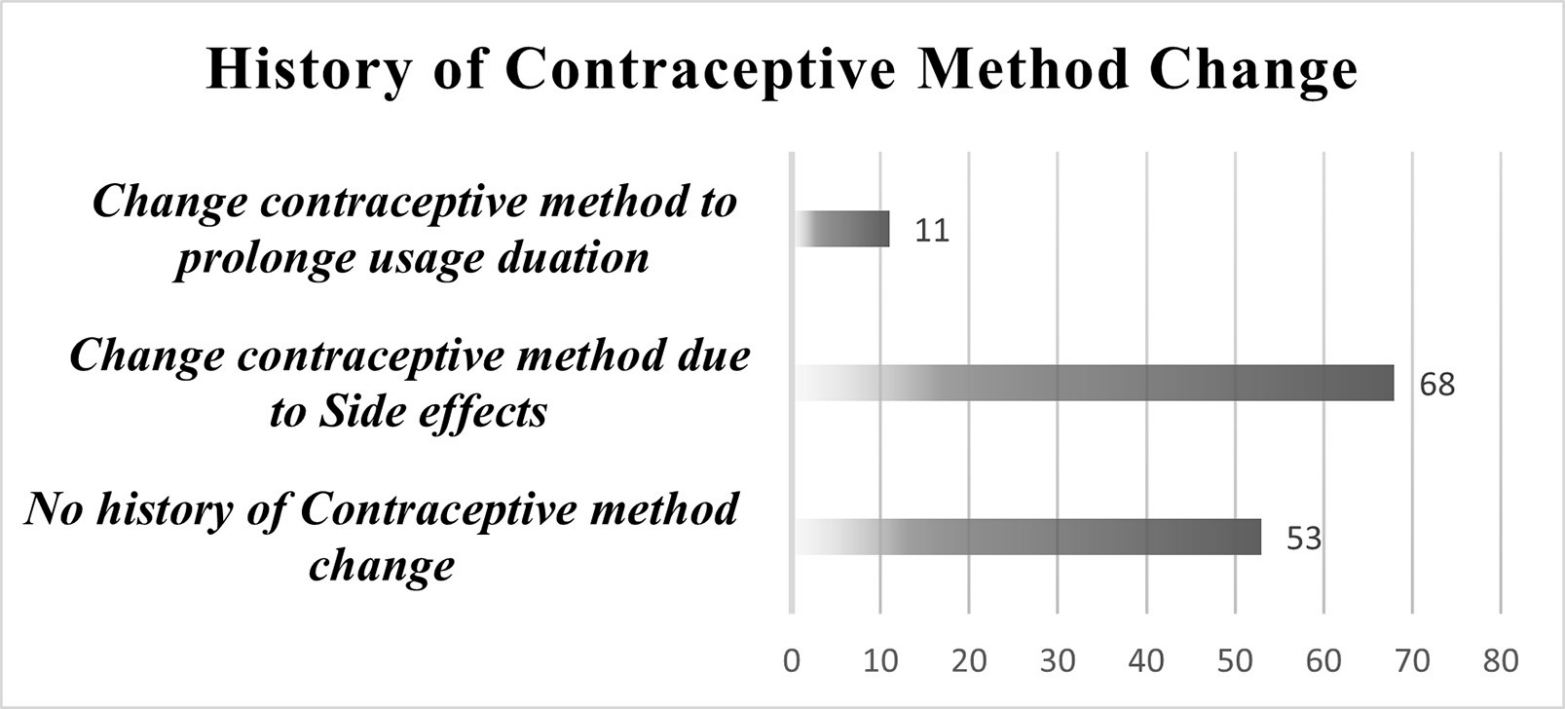
Previous history of changing hormonal contraceptive method.

From a total of 132 individuals enrolled as HC-users in the present study, 68 (51.51%) participants changed their initial contraceptive method due to side effects. On the other hand, 53 (32.5%) are on the same method since the initiation of contraceptive method (**Fig 3**) and (**Table 6**).

**Table 6:** Effect of changing hormonal contraceptive method on liver function tests.

Parameters	HC Users Who Never Change Their Method (n = 53)	Participants changed their method due to side effects (n = 68)	HC users who changed their method to prolong duration (n = 11)	P-value
	x±SD	x±SD	x±SD	
**AST (IU/L)**	50.45 (±16.79)^b^	46.44 (±13.00)	34.72 (± 6.14) ^a^	0.004*
**ALT (IU/L)**	38.64 (± 14.31) ^b^	35.42 (±13.51)	24.81 (± 4.11) ^a^	0.008*
**ALP (IU/L)**	66.01 (± 14.76)	62.60 (± 14.18)	55.0 (± 15.68)	0.06
**T- Bilirubin (mg/dl)**	0.72 (± 0.24)	0.67 (±0.21)	0.60 (± 0.11)	0.20
**D- Bilirubin (mg/dl)**	0.16 (± 0.07)	0.14 (± 0.05)	0.13 (± 0.06)	0.19
**GGT (IU/L)**	47.92 (± 25.02)	49.72 (± 24.68)	30.18 (±6.88) ^a^	0.04*

* Statistically significant difference in One way ANOVA

a-b Superscript letters represent statistical significance in post hoc Bonferroni post hoc test

“T-Bilirubin”–Total Bilirubin “D-Bilirubin”–Direct Bilirubin

a- p <0.05 when Participant change HC to prolong HC compared to HC user change due to side effects of the drug (One-way ANOVA/Bonferroni post hoc test)

b- p <0.05 when group who never change a method compared to HC user change due to prolong the time of HC use (One-way ANOVA/Bonferroni post hoc test)

Hormonal contraceptive users who never change their contraceptive method were observed to have a statistically significant (P<0.05) higher mean serum level of AST, ALT, and GGT than participants who change their contraceptive method to prolong the usage duration. On the other way around, HC-users who change HC due to side effects were observed to have a statistically significant (p<0.05) higher mean serum level of Serum AST and ALT (**Table 6**).

### Magnitude of liver function test abnormalities

The overall prevalence of liver abnormalities among the HC-user group for at least one LFT parameter was 35 (26.52%). From HC user groups, the prevalence of hypoproteinemia was found to be 28 (21.21%) and abnormal total and direct bilirubin 4 (3.03%) and 5 (3.79%) respectively. Hypoalbuminemia was found on 3 (2.27%) of HC users.

The total prevalence of liver abnormalities among control group for at least one LFT parameter was 37 (28.03%). Among Controls, the prevalence of hypoproteinemia was found to be 37 (28.03%).

## Discussion

A statistically significant higher mean value of AST, ALT, and ALP among HC-users than the non-user control groups in the present study was in agreement with the findings from previous studies conducted in Egypt, Nigeria, and Iraq [37, 39–42]. This observation could be attributed to the induction of liver enzymes associated with the administration of HCs; this may attributed to the effect of the Ethinylestradiol (EE) in HCs to increase the metabolic activities of the hepatocytes; larger doses of HCs cause adaptative changes by increasing the level of metabolic enzymes [7]. This premise is also supported by previous animal model experimental studies, which state that HCs induce alteration of enzymatic liver function tests in animal models [12]. However, opposing result was observed regarding the level of AST in study conducted in America [43]. This difference may be attributed to the dose difference and the type of HCs assessed; earlier generations of HC contained different forms of EE in structure and dose [44].

The significant elevation of ALT in the present study is in line with previous studies conducted in Poland, Iraq, and Iran [17, 30, 45]. This may be attributed to the rise in hepatic cell damage induced by EE. When the liver is compromised, liver enzymes leak enzymes into the circulating system causing increased activity. However, opposing result was obtained regarding the level of ALP, from previous studies from Nigeria, Iraq, France, and America [43, 46, 47] reported that HC use significantly reduced the level of ALP. This can also be explained by the fact that ALP derived mainly from liver and bone with approximately equal properties and the total effect of estrogen on bone. Estrogen is known inhibitor of the potential for Parathyroid hormone (PTH), particularly in its function. In women the using of HC containing estrogen, the PTH activity is inhibited and ALP is significantly reduced [48].

In the present study, the level of GGT is significantly higher among HC users than controls; this finding is consistent with the results obtained from France, Italy, and Poland; in their studies, an increase in GGT was observed to be favorably linked with HC use and an indicator of oxidative homeostasis disturbance in the liver microenvironment [17, 18, 49]. This is due to the triggering effect of HC on the occurrence of oxidative stress (OS). OS occurred due to the need to metabolize the hormone load from the HC with the help of liver enzymes. Both progestins and EE are subject to sulfate and glucuronide conjugation processes that require a high energy input. This reduction in energy availability can compromise the regeneration of reduced glutathione (GSH) from oxidized glutathione (GSSG), as well as the very availability of GSH, which is used to eliminate some metabolites [18].

The present study revealed that HC users showed a significant elevation of the mean total and direct bilirubin; this finding concurred with the studies form Egypt and Nigeria [29, 50]. This significant increase in the mean value of total bilirubin is suggestive of hepatobiliary complications, which are most likely induced by the prolonged use of HCs with the subsequent inhibition of the hepatic excretory function, thus leading to increased serum bilirubin [51]. However, opposing result was obtained from the study conducted in France, America, and Iraq [43, 47, 49]; these studies reported no significant difference in the mean value of serum total bilirubin; this difference may arise from the difference in sample size and study design, as most of these studies are short-term prospective studies.

In the current study, the mean value of total protein and albumin shows no statistical difference between HC users and controls; however, opposing results were obtained from the previous study from France [49] in which oral contraceptive pills (OC) were associated with a reduction in the level of serum albumin. This variation may be attributed to the differences in behavioral and diet characteristic among the study participants.

The mean serum levels of AST, ALT and ALP in the current study were 47.07 (± 14.79), 35.83 (± 13.78) and 63.34 (±14.74) respectively; the result concur with reports from Nigeria [37, 46] and Iraq [39]. However, this result is higher than the reports obtained from Nigeria [52], Egypt [50], and Poland [17]. The difference could be attributed to differences in participants behavioral characteristics from the current study. Correspondingly, the mean serum value of GGT in the present study was 47.37 (± 24.32); this value is in agreement with the study from Poland [17]. A higher value of GGT is a stronger predictor of disease risk than other symptoms. On the other hand, this result is higher than the report obtained from France [49]. The variation may arise from the difference in study design and behavioral characteristics of participants in the two studies.

In the current study, DMPA use was associated with a statistically significant higher mean value for ALT, ALP, total and direct bilirubin. This result is in agreement with the studies from Nigeria [37, 40], which report DMPA use is associated with liver alteration of liver integrity and functionality. This is because of the effect of EE content of the HCs on the metabolic activities of the hepatocytes and the induction of hepatic enzymes that accompany the administration of injectable contraceptives [40].

The present study revealed that long-term use of HCs (for >42 months/>3.5 years) causes a significant lower level in the mean value of ALP, this decrement was consistent with the study from Iraq in which long-term contraceptives showed a reduced level of ALP [30]. This may be due to the inhibition of recruitment of bone cells by EE, in which HC was associated with bone loss and a subsequent lower level of ALP [53].

On the present study the mean values of ALT, GGT and total bilirubin were found to be statistically significantly (higher in Combined oral contraceptive users than Norplant/Implant and DMPA users’ this finding is consistent with studies conducted in Poland and India [17, 54]. This is due to a relatively higher dose of EE in COC than other contraceptive method. EE has been reported to generate free radicals by metabolic redox cycling between quinone and hydroquinone forms of EE [7]. Higher dose of EE also induces a clinically apparent cholestatic liver marked by a higher level of serum bilirubin. EE and particularly combinations of estrogens and progestins have been linked to episodes of marked serum ALT elevations without symptoms, jaundice, or cholestasis [31, 55].

## Conclusions

The use of hormonal contraceptives induced changes in liver function tests; the activity of liver serum enzymes and the mean value of total and direct bilirubin were higher among hormonal contraceptive users than respective controls. However, the mean levels of total protein and albumin were not different among the two groups. This change has been observed to be associated with the class of hormonal contraceptives and the duration of HC use.

## Recommendations

It is better to conduct further studies with a larger sample size and equal or matched number of participants based on: menopausal status; using a longer follow up study design to investigate the effect of hormonal contraceptives on liver function tests and histological changes in the liver. It is better to do a follow up and/or continuous checkups for women on hormonal contraceptives for liver function tests, which would be helpful for early detection of adverse effects. It is also better to provide women with information regarding the signs of any related side effects, and prompt medical consultation and prior screening should be conducted before the initiation of the HC regime. The findings of the present study suggest the need for healthcare providers to undertake appropriate client profiling before recommending a particular class of hormonal contraceptives to women who go to initiate the family planning services in order to minimize adverse effects, especially for those who may have pre-existing conditions and It is better for individuals who are on hormonal contraceptives to follow healthy behavioral and lifestyle since these factors observed to reduce the occurrence of adverse effects associated with hormonal contraceptive utilization.

## Strength and limitation of the study

### Strength of the study

To the best of our searching efforts, it is the first study in Ethiopia to assess the liver function test parameters of hormonal contraceptive users, and it also paves the way for further studies. And in contrast to most of the previous studies, this study incorporated more biochemical liver function test parameters than any other previous study conducted. All laboratory test parameters were analyzed in the referral clinical chemistry laboratory of University of Gondar Comprehensive Specialized Hospital (UoGCSH), and all participants were screened for HBV, HCV, HIV/AIDS, and metabolic syndrome prior to inclusion in the study.

### Limitation of the study

One of the limitations of the study is that, even though we include the comparison control group, the cross-sectional nature of the study limits the ability to determine causal relationships between the use of hormonal contraception and the health outcomes considered. Due to the unavailability and cost of the assays, serum ALP was analyzed without identifying bone specific ALP. Moreover, we were unable to measure PTH, which is a better indicator of effect of estrogen in ALP value. Some chronic disease screening was not done; we took the data from medical history and participants’ medical charts. Some of the information, like the diet characteristics and, habits of smoking and alcohol consumption, was subjectively obtained from participants and not in-depth assessed.

## List of abbreviations

ALP: Alkaline Phosphates
ALT: Alanine aminotransferase
ANOVA: Analysis of Variance
AST: Aspartate Aminotransferase
BIL-D: Direct Bilirubin
BIL-T: Total Bilirubin
BMI: Body Mass Index
BP: Blood Pressure
CI: Confidence Interval
DBP: Diastolic Blood Pressure
DMPA: Depot-Medroxyprogesterone Acetate
E_2_: Estradiol
EE: Ethinylestradiol
FBS: Fasting Blood Sugar
FDA: Federal Drug Administration
GGT: γ-glutamyl transferase
HBV: Hepatitis B Virus
HCs: Hormonal Contraceptives
HCV: Hepatitis C Virus
HC: Hormonal Contraceptives
HTN: Hypertension
LFTs: Liver function tests
NET: Norethisterone
OC: Oral Contraceptives
OS: Oxidative Stress
PTH: Parathyroid Hormone
SBP: Systolic Blood Pressure
WHO: World Health Organization
